# A palmitoylation-related signature predicts prognosis and immune microenvironment features in diffuse large B-cell lymphoma

**DOI:** 10.3389/fimmu.2026.1907503

**Published:** 2026-09-22

**Authors:** Jie Zhang, Xiaobing Miao, Hao Yang, Ying Zhou, Yijing Jiang, Qian Shen, Xiaohong Xu

**Affiliations:** 1 Department of Oncology, Affiliated Tumor Hospital of Nantong University, Nantong, Jiangsu, China; 2 Department of Pathology, Affiliated Tumor Hospital of Nantong University, Nantong, Jiangsu, China; 3 Department of Pathophysiology, School of Medicine, Nantong University, Nantong, Jiangsu, China

**Keywords:** diffuse large B-cell lymphoma, machine learning, palmitoylation, prognosis prediction, tumor immune microenvironment

## Abstract

**Background:**

Diffuse large B-cell lymphoma (DLBCL) is the most common aggressive lymphoma and remains highly heterogeneous in prognosis. Palmitoylation, an important post-translational modification, has been implicated in tumor biology, but its role in DLBCL, particularly in relation to the immune microenvironment, remains unclear.

**Methods:**

Transcriptomic and clinical data from 706 patients with DLBCL were obtained from the Gene Expression Omnibus database. Prognosis-associated palmitoylation-related genes were identified by differential expression and univariate Cox regression analyses. An optimal prognostic model was developed by comparing 101 machine learning algorithm combinations and was further integrated with clinical variables to construct a nomogram. The tumor immune microenvironment was evaluated using immune infiltration, immune function, immune checkpoint, and Tumor Immune Dysfunction and Exclusion analyses. Weighted gene co-expression network analysis (WGCNA) and Mendelian randomization (MR) were performed to identify key genes, followed by experimental validation.

**Results:**

A 33-gene palmitoylation-related signature (PRS) was established and evaluated across multiple cohorts. PRS was an independent prognostic factor of DLBCL and showed consistent prognostic performance across datasets. Low-risk patients showed higher immune cell infiltration and more active immune function, whereas high-risk patients showed a greater predicted potential to respond to immune checkpoint inhibitor therapy. A nomogram integrating PRS with clinical characteristics also showed favorable predictive accuracy. In addition, two palmitoylation-related molecular subtypes with distinct transcriptomic features, survival outcomes, and immune microenvironment characteristics were identified in DLBCL. *CEACAM3* was prioritized as a key candidate gene based on integrated WGCNA and MR analyses, and functional experiments showed that *CEACAM3* knockdown suppressed proliferation and invasion and promoted apoptosis, whereas *CEACAM3* overexpression enhanced proliferation and invasion, reduced apoptosis, and promoted tumor growth.

**Conclusion:**

The PRS captures prognostic heterogeneity and distinct immune microenvironment features in DLBCL. PRS may provide a useful tool for risk stratification, and *CEACAM3* may serve as a potential biomarker and therapeutic target in DLBCL.

## Introduction

Diffuse large B-cell lymphoma (DLBCL) is the most common pathological subtype of non-Hodgkin lymphoma, accounting for approximately 40% of lymphoid malignancies and exhibiting marked morphological and molecular heterogeneity (1, 2). The standard first-line treatment is chemoimmunotherapy, most commonly the R-CHOP regimen (2, 3). Although targeted therapies have been developed and many patients respond well to treatment, approximately 30%-40% relapse and about 10% of newly diagnosed patients remain difficult to treat (4, 5). These differences in treatment outcome are closely related to the high heterogeneity of DLBCL. Therefore, a deeper understanding of its molecular heterogeneity is essential for precise treatment and improved prognosis. With advances in sequencing technologies and molecular biology, multiple abnormalities in key signaling pathways involved in DLBCL tumorigenesis, metastasis, and chemotherapy response have been identified. In this context, multigene expression-based risk models have shown prognostic value and may provide a basis for individualized treatment strategies (6–8). However, their systematic construction, validation, and clinical application in DLBCL remain limited. In addition to tumor-intrinsic molecular alterations, accumulating evidence indicates that the tumor immune microenvironment (TIME), a key component of the broader tumor microenvironment (TME), plays a critical role in DLBCL progression, therapeutic response, and clinical outcomes. The composition and functional state of infiltrating immune cells vary substantially among patients and may contribute to immune escape, treatment resistance, and survival heterogeneity. Therefore, integrating molecular stratification with immune microenvironmental features may provide a more comprehensive framework for understanding DLBCL heterogeneity and improving risk assessment.

Palmitoylation is a widespread and dynamic post-translational modification, and its reverse process is termed depalmitoylation (9, 10). It has been shown to regulate the membrane localization, stability, and interactions of key oncogenic proteins, thereby contributing to tumor initiation and progression (11). In prostate and colorectal cancers, palmitoylation promotes tumor proliferation and invasion by regulating pathways such as Wnt/β-catenin and p38/MAPK (12–14). In breast cancer, it is also associated with chemoresistance (15). Notably, palmitoylation has been implicated in the development and drug resistance of DLBCL (16, 17). Therefore, constructing a risk prediction model based on palmitoylation-related genes (PRGs) is of clear clinical and scientific interest. Notably, emerging studies suggest that palmitoylation is also involved in the regulation of immune signaling, membrane receptor function, and cell-cell interactions, indicating that palmitoylation-associated molecular states may influence not only tumor behavior but also the immune contexture of the TME. Moreover, the prognostic relevance of palmitoylation-related signatures (PRSs) has been reported in several malignancies, including glioblastoma (18), hepatocellular carcinoma (19), and gastric cancer (20). Nevertheless, whether PRS can be used for prognostic stratification in DLBCL, and how they relate to the immune landscape of this disease, remain insufficiently characterized.

In this study, we systematically investigated the role of PRGs in DLBCL and constructed a PRG-based prognostic risk model by integrating 10 machine learning algorithms into 101 model combinations. We also prioritized CEACAM3 as a key candidate gene through integrated weighted gene co-expression network analysis (WGCNA) and Mendelian randomization (MR) analyses, analyzed its biological and prognostic significance in DLBCL, and further validated its function experimentally. Collectively, our findings may provide new strategies for risk stratification and individualized treatment in DLBCL and characterize the immunological heterogeneity associated with PRS.

## Materials and methods

### Data collection and processing

Transcriptome and clinical data of DLBCL were obtained from the Gene Expression Omnibus (GEO) database, including four datasets: GSE56315, GSE10846, GSE11318, and GSE53786. Among these, GSE56315 (55 tumor samples and 33 normal samples) was used to identify differentially expressed genes (DEGs) between tumor and normal tissues. GSE10846 was used as the training cohort, whereas GSE11318 and GSE53786 served as validation cohorts for prognosis model development and validation. To minimize potential confounding from early deaths unrelated to tumor progression, only patients with an overall survival of >30 days were included in the construction and validation of the prognostic model, resulting in 399 patients from GSE10846, 193 from GSE11318, and 114 from GSE53786. Microarray probes were mapped to gene symbols according to the corresponding platform annotation files. Probes without gene annotation were removed, and when multiple probes mapped to the same gene, their expression values were averaged to obtain a single gene-level expression value. Transcriptome data within each dataset were normalized, and batch effects among these four datasets were adjusted using the *ComBat* function implemented in the “*sva*” R package (21) (**Supplementary Figures S1A, B**). In addition, 3,680 PRGs were retrieved from the GeneCards database using the keyword “Palmitoylation” (https://www.genecards.org/) (**Supplementary Table 1**). This GeneCards-derived gene set was used as a broad discovery set and included genes associated with palmitoylation based on database annotation and literature evidence.

### DEGs identification and screening of PRGs for prognosis

In GSE56315, DEGs between DLBCL and normal tissues were identified using the “*limma*” R package (22). After multiple testing correction, genes with an FDR < 0.05 and |log_2_FC| > 0.585 were considered significantly differentially expressed. Gene Ontology (GO) and Kyoto Encyclopedia of Genes and Genomes (KEGG) enrichment analyses of the identified DEGs were subsequently performed using the “*Hs.eg.db*” and “*clusterProfiler*” R packages (23), with the top 5 GO terms and top 20 significant KEGG pathways presented, respectively. The intersection between DEGs and the PRG set was defined as palmitoylation-related differentially expressed genes (PDGEs). In the training cohort, univariate Cox regression analysis was then performed to investigate the association between PDGE expression and overall survival in patients with DLBCL. PDGEs significantly associated with prognosis at *P* < 1×10^–4^ were retained as prognostic PDGEs for subsequent analyses.

### Identification and characterization of palmitoylation-related molecular subtypes

Based on the expression matrix of prognostic PDGEs, non-negative matrix factorization (NMF) clustering was performed using the “*NMF*” R package (24) to identify distinct palmitoylation-related molecular subtypes of DLBCL. The optimal number of subtypes was determined based on the cophenetic coefficient, with *K* = 2 selected for subsequent analyses. Principal component analysis (PCA) was performed to visualize the transcriptomic distribution of the identified subtypes. Kaplan-Meier survival analysis was subsequently used to compare overall survival between the subtypes. Gene set variation analysis (GSVA) was performed using the “*GSVA*” R package to compare differences in pathway activity between the subtypes. In addition, immune cell infiltration was estimated using CIBERSORT, and the relative abundance of 22 immune cell types was compared between the subtypes to characterize differences in the tumor immune microenvironment.

### Development and validation of a prognostic signature based on PRGs

Based on 99 prognostic PDGEs, we developed a prognostic signature for DLBCL using an integrated machine learning strategy. Specifically, 10 machine learning algorithms were systematically integrated: least absolute shrinkage and selection operator regression (Lasso, “*glmnet*” package (25)), ridge regression (Ridge, “*glmnet*” package), stepwise Cox regression (stepwise Cox, “*stats*” package), CoxBoost (“*CoxBoost*” package (26)), random survival forest (RSF, “*RandomForestSRC*” package (27)), elastic net regression (Enet, “*glmnet*” package (25)), Cox partial least squares regression (plsRcox, “*plsRcox*” package (28)), supervised principal component regression (SuperPC, “*superpc*” package (29)), gradient boosting machine (GBM, “*gbm*” package (30)), and survival support vector machine (survival-SVM, “*survivalsvm*” package (31)). Using these algorithms, a total of 101 candidate models were systematically generated, including single-algorithm models and sequential combinations of feature-selection and model-construction algorithms. For combined models, the first algorithm was used to select prognostic features from the candidate PDGEs in the training cohort, and the selected genes were subsequently used to establish the final prognostic model using the second algorithm. For each candidate model, individual risk scores were calculated for patients in the training and validation cohorts, and model performance was evaluated using Harrell’s concordance index (C-index). The average C-index across the training and validation cohorts was used as the primary criterion for model comparison, and all candidate models were ranked according to this value. The model with the highest average C-index was considered the optimal prognostic model. Finally, the StepCox[both] + plsRcox model achieved the best predictive performance and was selected to construct the PRS. In this model, bidirectional stepwise Cox regression was first applied for feature selection, followed by partial least squares Cox regression to establish the PRS. The PRS score was calculated as follows:


$$\text{Risk Score}=\sum_{i=1}^{n}coef_{i}*\;EXP_{i}$$

Where *coef*_i_ represents the regression coefficient of the ith PDGE, *EXP*_i_ represents the relative expression level of the ith PDGE, and *n* represents the total number of genes included in the signature. Based on the risk model formula, risk scores were calculated for each patient in the training and validation cohorts. Patients in the training cohort and each validation cohort were stratified into high- and low-risk groups according to the median PRS within the corresponding cohort. The median cutoffs were -12.3094, -12.2898, and -12.2966 for GSE10846, GSE11318, and GSE53786, respectively. The “*survival*” and “*timeROC*” R packages (32) were used to generate Kaplan-Meier survival curves and time-dependent receiver operating characteristic (ROC) curves, respectively, for the high- and low-risk groups. Furthermore, to benchmark the predictive performance of the PRS, we compared it with 10 previously published DLBCL prognostic gene signatures and the conventional International Prognostic Index (IPI) in the GSE10846 cohort. For each published signature, its coefficients were re-estimated using multivariable Cox regression. Individual risk scores were then calculated, and patients were stratified into high- and low-risk groups according to the median risk score of each refitted signature.

### Nomogram construction

To evaluate the prognostic value of the PRS in combination with clinical characteristics, age, gender, ECOG score, stage, and extranodal sites were included in subsequent analyses; samples with missing clinical variables were excluded from analysis. Differences in clinical characteristics between the high- and low-risk groups were first compared. Clinical characteristics and PRS were then included in univariate and multivariate Cox regression analyses to confirm their prognostic value. An overall survival nomogram was constructed using the “*rms*” R package (33). The “*survival*”, “t*imeROC*” and “*pec*” R packages (34) were used to generate calibration plots, ROC curves, and time-dependent C-index curves to evaluate nomogram performance.

### Analysis of the TME in relation to PRS of DLBCL

The association between PRS and the TME in DLBCL was investigated using integrated bioinformatic approaches. Specifically, CIBERSORT was used to estimate the relative infiltration abundance of 22 immune cell subsets, while ESTIMATE was applied to calculate stromal and immune scores and to infer tumor purity. To further characterize immune-related features, 29 immune-related signatures were obtained from a previously published study (35) and used to compare differences between the high- and low-risk groups. The expression levels of 47 immune checkpoint genes were also compared between the two risk groups, and the complete gene list is provided in **Supplementary Table 2**. In addition, Tumor Immune Dysfunction and Exclusion (TIDE, http://tide.dfci.harvard.edu) analysis was performed to assess the potential for tumor immune evasion.

### Drug sensitivity analysis

To predict drug sensitivity in different risk groups, the GDSC database and the “*oncoPredict*” R package (36) were used. Predicted half-maximal inhibitory concentration (IC50) values were calculated for 198 drugs. Candidate drugs were identified based on both differences in predicted IC50 values between the high- and low-risk groups and correlations between IC50 values and PRS scores.

### Weighted Gene Co-expression Network Analysis (WGCNA)

To identify core genes associated with the two risk groups, WGCNA was performed on the DEGs. First, the top 50% of genes with the highest median absolute deviation were selected to construct the co-expression network. The target value of the scale-free topology fitting index R^2^ was set at 0.9, and the *pickSoftThreshold* function was used to determine the optimal soft-thresholding power β (β = 10) for network construction, thereby establishing a gene co-expression network with scale-free topology. Finally, module-trait correlation analysis was performed to identify the key gene modules most strongly associated with DLBCL risk groups.

### Mendelian randomization (MR) analysis

To further explore the causal relationship between PRGs and DLBCL, MR analysis of PDGEs was performed using the “*TwoSampleMR*” R package (37). Gene eQTL data derived from the eQTLGen whole-blood dataset were obtained from the MRC-IEU GWAS database. This resource was selected because of its large sample size and broad gene coverage, which provided sufficient statistical power for instrumental variable selection. The summary statistics for the outcome “Diffuse large B-cell lymphoma” were obtained from the FinnGen database (version R12). For robust MR analysis, single-nucleotide polymorphisms (SNPs) were selected as instrumental variables (IVs) if they were associated with the exposure (*P* < 5×10^-6^) and remained independent after linkage disequilibrium pruning (r^2^ < 0.001, distance > 10,000 kb). This threshold was used to increase the availability of IVs and enable exploratory MR analysis across a broader range of PDGEs. The F-statistic was calculated for each SNP, and only SNPs with an F-statistic > 10 were retained. Steiger filtering was further performed to ensure that the retained SNPs were consistent with the hypothesized causal direction from gene expression to DLBCL. Five methods, including MR-Egger, weighted median, inverse-variance weighted (IVW), simple mode, and weighted mode, were used for comprehensive analysis to explore the potential causal relationship between PDGEs and DLBCL. The IVW method was used as the primary MR analysis, with nominal *P* < 0.05 used for initial screening. Benjamini-Hochberg FDR correction was subsequently applied across the tested PDGEs to account for multiple comparisons. To ensure the reliability of MR estimates, we assessed heterogeneity using Cochran’s Q test and directional horizontal pleiotropy using the MR-Egger intercept test. Potential pleiotropic outliers were further identified using MR-PRESSO.

### Cell lines and cultures

The human DLBCL cell lines SU-DHL-4 and TMD8 were obtained from Shanghai Zhong Qiao Xin Zhou Biotechnology (China) and Wuhan Pricella Biotechnology (China), respectively. Both cell lines were cultured in RPMI-1640 medium (ZM0982, Shanghai Zhong Qiao Xin Zhou Biotechnology, China) supplemented with 10% fetal bovine serum (FBS). Cells were maintained in a humidified incubator at 37 °C with 5% CO_2_ and passaged when they reached an appropriate density during the logarithmic growth phase.

### Cell transfection

The epiCRISPR/Cas9 plasmid expressing *CEACAM3*-targeting sgRNAs was constructed by Shanghai Transheep (China). The cloned sgRNA sequences were as follows: sgRNA-1, CAGGACCCCGGTCACGATGC; sgRNA-2, AGAAGTTTAGAAGTGAGGCT. To produce lentiviral particles, 293T cells were co-transfected with the *CEACAM3*-targeting constructs and the corresponding packaging plasmids using Lipofectamine™ 3000 Transfection Reagent (Invitrogen, USA) according to the manufacturer’s instructions. The viral supernatants were collected and used to transduce SU-DHL-4 cells. Stable cell populations were selected with puromycin, and transduction efficiency was assessed by western blotting.

For *CEACAM3* overexpression, TMD8 cells were transfected with a FLAG-tagged human *CEACAM3* expression plasmid purchased from GeneChem Co., Ltd. (Shanghai, China) using Lipofectamine™ 3000 Transfection Reagent (Invitrogen, USA) according to the manufacturer’s instructions. Cells transfected with the corresponding empty vector served as the control. At 48 h after transfection, the cells were subjected to puromycin selection for 7 days to establish stable *CEACAM3*-overexpressing and control cell populations. *CEACAM3* overexpression efficiency was confirmed by western blotting before subsequent *in vitro* functional assays and establishment of the subcutaneous xenograft model.

### Western blot

Cells were harvested and lysed on ice in RIPA buffer supplemented with PMSF (R0020, Solarbio, China) for 30 min. After centrifugation at 4 °C, protein concentrations were determined using a BCA Protein Assay Kit (C503021, Sangon Biotech, China) according to the manufacturer’s protocol. Equal amounts of protein were separated by 10% SDS-PAGE and transferred onto PVDF membranes (IPVH00010, Millipore, USA). Membranes were blocked with 5% skim milk in TBST for 1 h at room temperature and incubated with primary antibodies overnight at 4 °C. After three washes with TBST, membranes were incubated with HRP-conjugated secondary antibodies for 1 h at room temperature. Immunoreactive bands were detected using enhanced chemiluminescence substrate (BL520B, Biosharp, China) and visualized with a chemiluminescence imaging system. Primary antibodies were anti-CEACAM3 (DF2694, Affinity, China) and anti-Vinculin (sc73614, Santa Cruz, USA) and anti-Flag (F3165, Sigma, USA).

### Cell proliferation assay

Cell proliferation of SU-DHL-4 cells and TMD8 cells were evaluated using a Cell Counting Kit-8 (CCK-8) assay (A311-01, Nanjing Vazyme Biotech, China). After transfection, cells were seeded into 96-well plates at 2×10^4^ cells/mL (200 μL/well) and incubated for 24 h at 37 °C with 5% CO_2_. Then, 20 μL CCK-8 solution was added to each well and incubated for another 4 h. Absorbance at 450 nm was measured using a microplate reader.

### Transwell invasion assay

Cell invasive ability was assessed using 24-well Transwell plates with 8.0-µm pore inserts (cat. no. 3422; Corning, USA). Matrigel matrix (cat. no. 354234; Corning, USA) was thawed overnight at 4 °C and diluted 1:6 with serum-free culture medium. Subsequently, 50 µL of the diluted Matrigel was added to the upper chamber of each Transwell insert and incubated at 37 °C for 1–2 h to allow gel polymerization. A total of 2 × 10^5^ cells were resuspended in 200 µL serum-free medium and seeded into the upper chamber. The lower chamber was filled with 600 µL complete medium supplemented with 10% FBS, which served as a chemoattractant. After incubation for 24–48 h at 37 °C in a humidified incubator containing 5% CO_2_, the medium in the lower chamber was collected and centrifuged at 1,000 rpm for 5 min. The resulting cell pellets were washed twice with phosphate-buffered saline (PBS), fixed with 4% paraformaldehyde for 15 min, and stained with 0.1% crystal violet staining solution (cat. no. G1063; Solarbio, China) for 20 min. After being washed twice with PBS, the cells were resuspended in 100 µL PBS. Then, 10 µL of the cell suspension was placed onto a glass slide and mounted with a coverslip. Invaded cells were photographed and counted in three randomly selected fields under a light microscope.

### TUNEL staining

Stable *CEACAM3* knockdown SU-DHL-4 cells, stable *CEACAM3* overexpressing TMD8 cells, and their corresponding control cells were collected and washed with PBS. Cells were fixed with 1% paraformaldehyde at 4 °C for 20 min, rinsed with PBS, and permeabilized with 0.2% Triton X-100 for 5 min at room temperature. TUNEL labeling was then carried out according to the manufacturer’s instructions (A113-01, Nanjing Vazyme Biotech, China). Fluorescence images were acquired using a fluorescence microscope, and the percentage of TUNEL-positive cells was calculated using ImageJ software in three randomly selected fields per sample.

### Subcutaneous xenograft model

Eight-week-old male NPG mice were purchased from Nantong Jingqi Biotechnology Co., Ltd. (China) and housed under specific pathogen-free conditions. A total of 12 mice were assigned to the control and *CEACAM3* overexpression groups, with six mice in each group. Stable control and *CEACAM3* overexpressing TMD8 cells in the logarithmic growth phase were harvested, washed, and resuspended in RPMI-1640 medium. For each mouse, 1 × 10^7^ cells in 50 μL of medium were mixed with 50 μL of Matrigel, resulting in a final injection volume of 100 μL. The cell suspension containing Matrigel was subcutaneously injected into the right axillary region of each mouse.

Tumor growth was monitored throughout the experimental period. Tumor length and width were measured regularly using a digital caliper, and tumor volume was calculated using the following formula: tumor volume = length × width²/2. Mice were euthanized at 15 days after inoculation by CO2 asphyxiation with a chamber flow rate of 30% of the chamber volume per minute, followed by cervical dislocation. The tumor tissues were excised and fixed in 4% paraformaldehyde for further analysis. Ki-67 immunohistochemistry (9129s, Cell Signaling Technology, USA) was performed.

### Statistical analysis

All statistical analyses and visualizations were performed using R software (version 4.3.1). Differential expression analysis was based on a linear model with empirical Bayes moderation. Correlations between variables were evaluated using Spearman correlation coefficients. Continuous variables were compared using Student’s t-test or the Wilcoxon rank-sum test, as appropriate. Unless otherwise specified, a two-sided *P* < 0.05 was considered statistically significant. The overall workflow is shown in **Figure 1**.

**Figure 1:**
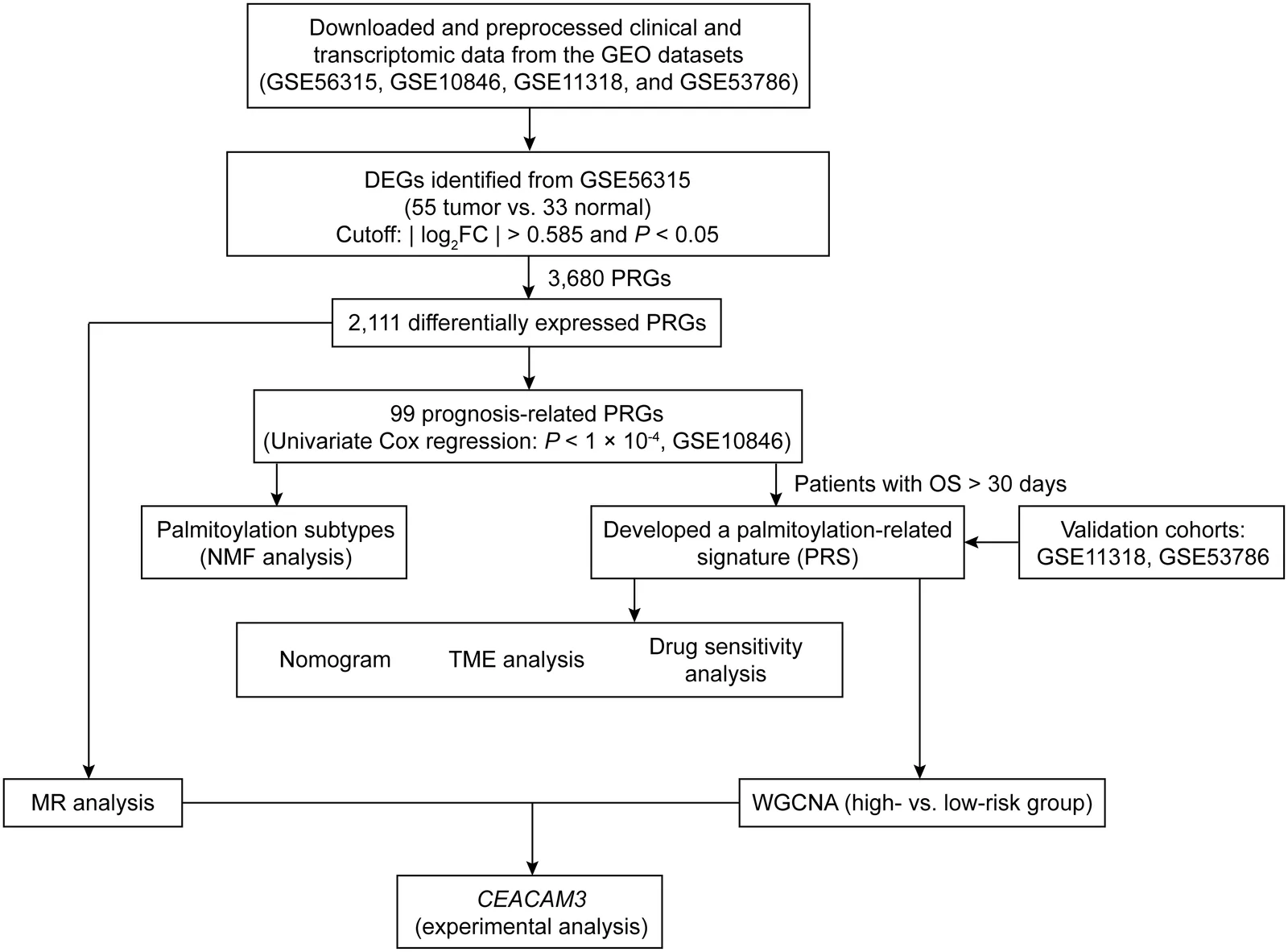
Flowchart of the study.

## Results

### Identification of prognostic PRGs

Differential expression analysis of the GSE56315 dataset identified 16,236 DEGs between DLBCL and normal tissues, including 8,094 upregulated and 8,142 downregulated genes (**Figures 2A, B**). To investigate the biological functions of DEGs, GO and KEGG enrichment analyses were performed. GO analysis showed that the DEGs were mainly enriched in cytokine-mediated signaling pathways, collagen-containing extracellular matrix, and extracellular matrix structural constituent functions (**Figures 2C, D**). KEGG analysis further indicated that the DEGs were mainly enriched in pathways related to intercellular communication, cell movement and adhesion, and signal transduction (**Figures 2E, F**). Detailed results are provided in **Supplementary Tables 3** and **S4**. These findings suggest that the DEGs may be involved in multiple biological processes relevant to DLBCL progression.

**Figure 2:**
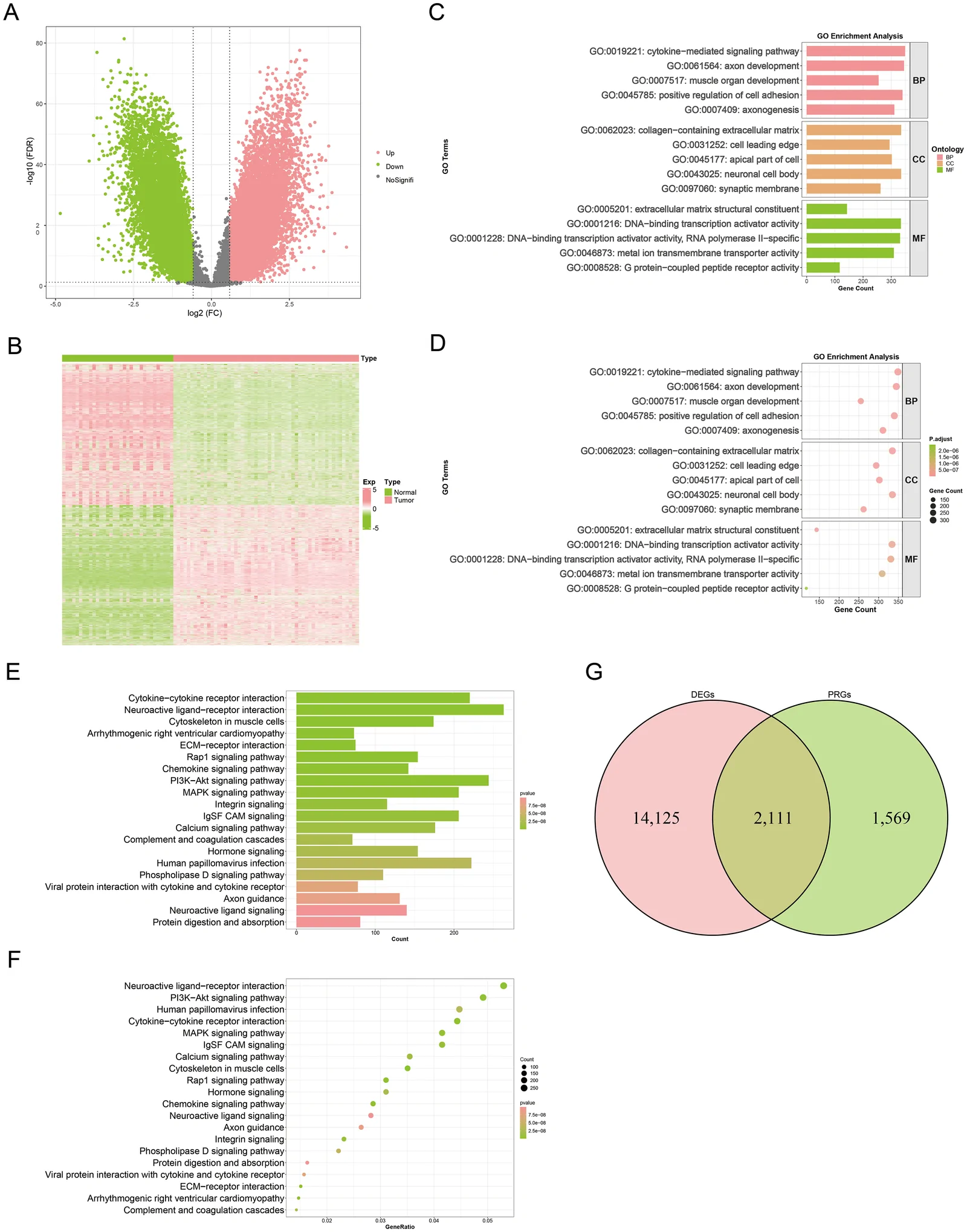
Identification and functional enrichment analysis of DEGs. **(A)** Volcano plot of DEGs between DLBCL tumor and normal tissues. **(B)** Heatmap of DEGs. **(C, D)** Bar charts and bubble plots of GO enrichment analysis. **(E, F)** Bar charts and bubble plots of KEGG enrichment analysis. **(G)** Venn plot showing the intersection between DEGs and PRGs.

To further explore the role of PRGs in DLBCL, the DEGs were intersected with the known PRG set, yielding 2,111 PDGEs (**Figure 2G**). In the GSE10846 dataset, univariate Cox regression analysis of these PDGEs identified 99 genes significantly associated with overall survival (*P* < 1×10^-4^, **Supplementary Table 5**, **Supplementary Figure S2**).

### Identification of palmitoylation subtypes in DLBCL

To further characterize the heterogeneity of DLBCL, NMF clustering was performed based on the 99 prognostic PDGEs. The clustering heatmap (**Figure 3A**, **Supplementary Figure S3**) showed that DLBCL samples could be divided into two molecular subtypes, cluster 1 (C1) and cluster 2 (C2), with distinct gene expression patterns between the two groups (**Figure 3B**). Principal component analysis further demonstrated distinct transcriptomic distributions between the two subtypes (**Figure 3C**). In addition, overall survival differed significantly between the two subtypes, with patients in the C2 subgroup showing a better prognosis than those in the C1 subgroup (**Figure 3D**).

**Figure 3:**
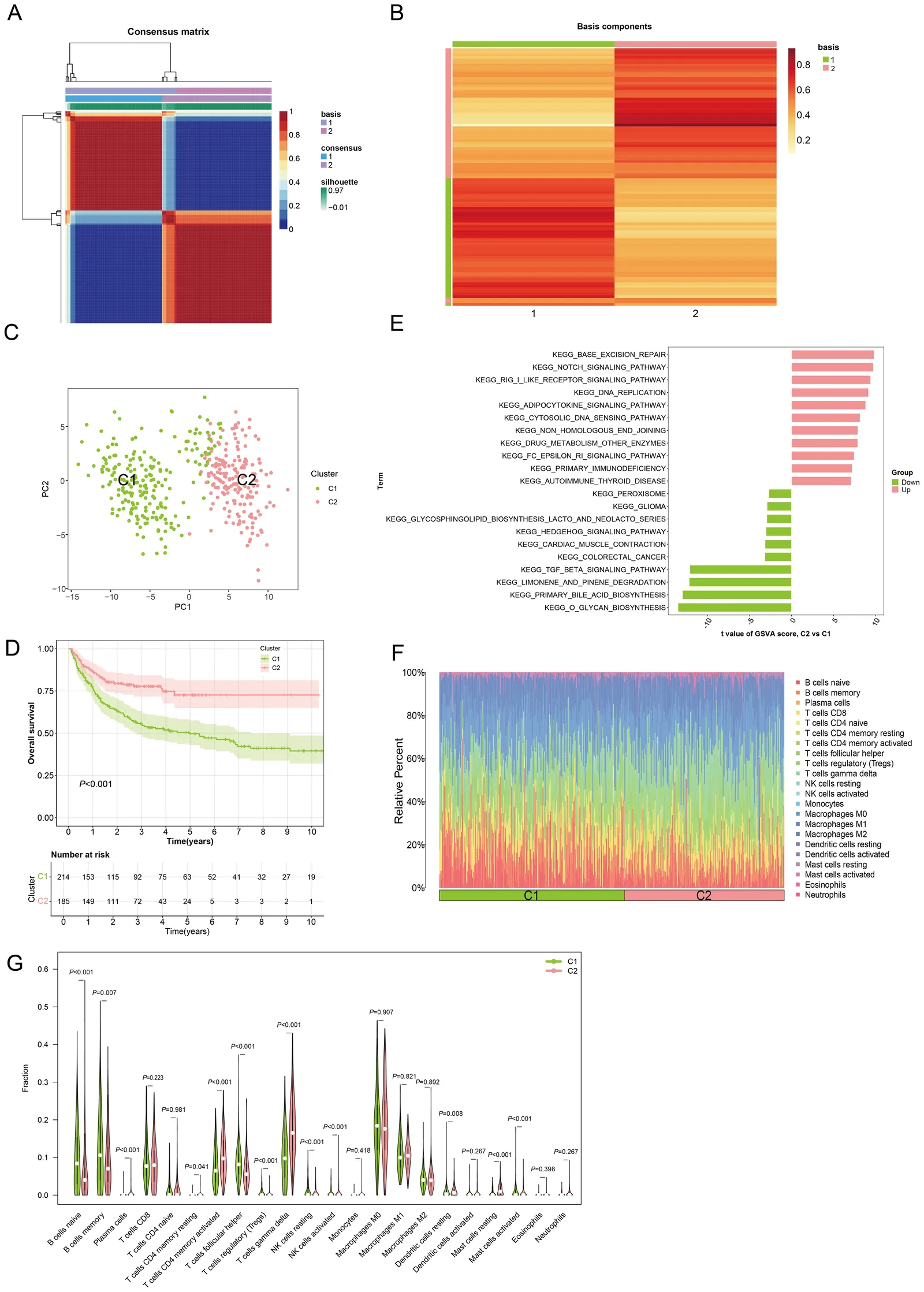
Identification and characterization of palmitoylation subtypes. **(A)** Heatmap of NMF clustering (*K* = 2). **(B)** Heatmap of gene expression between the two subtypes. **(C)** PCA of C1 (n=214) and C2 (n=185). **(D)** Kaplan-Meier survival curve comparing overall survival between the two subtypes. **(E)** GSVA between the two subtypes. **(F)** Stacked bar plot of immune cell infiltration in the two subtypes. **(G)** Differences in infiltration levels of 22 immune cell types between the two subtypes.

GSVA results showed that the C1 subgroup was mainly enriched in oncogenic signaling pathways, metabolic processes, and core cellular signal transduction pathways, whereas the C2 subgroup was enriched in pathways related to genomic stability and adaptive immune responses (**Figure 3E**). These results indicated distinct biological characteristics between the two subtypes. Immune analysis further revealed significant differences in the TIME between the two subgroups. As shown in (**Figures 3F, G**), the C2 subgroup exhibited a more active immune infiltration state, with 13 immune cell types differing significantly between the two groups. Together, these results indicate that PDGE-defined DLBCL subtypes differ substantially in transcriptomic profiles, pathway activity, and the immune microenvironment, suggesting that palmitoylation-related molecular signatures may be associated with the biological heterogeneity of DLBCL.

### Construction of the PRS prognostic model

To identify the optimal prognostic model, 101 machine learning model combinations were systematically generated and evaluated. After comparing predictive performance across the training and validation cohorts, the StepCox[both] + plsRcox combination was selected as the optimal algorithm for constructing the PRS because it achieved the highest average C-index (**Figure 4A**). The C-index was 0.787 in the training cohort and 0.729 and 0.828 in the two validation cohorts (GSE11318 and GSE53786), respectively. The final PRS model comprised 33 prognostic PDGEs. Details of each gene and its corresponding regression coefficient, and the individual PRS values for each patient, are provided in **Figure 4B** and **Supplementary Tables 6**, **7**. Using the cohort-specific median PRS as the cutoff, patients were stratified into high- and low-risk groups, comprising 199 and 200 patients in GSE10846, 97 and 96 patients in GSE11318, and 57 and 57 patients in GSE53786, respectively. As the PRS score increased, the proportion of death events also increased across the three cohorts, accompanied by differences in the expression of model genes (**Figures 4C–E**).

**Figure 4:**
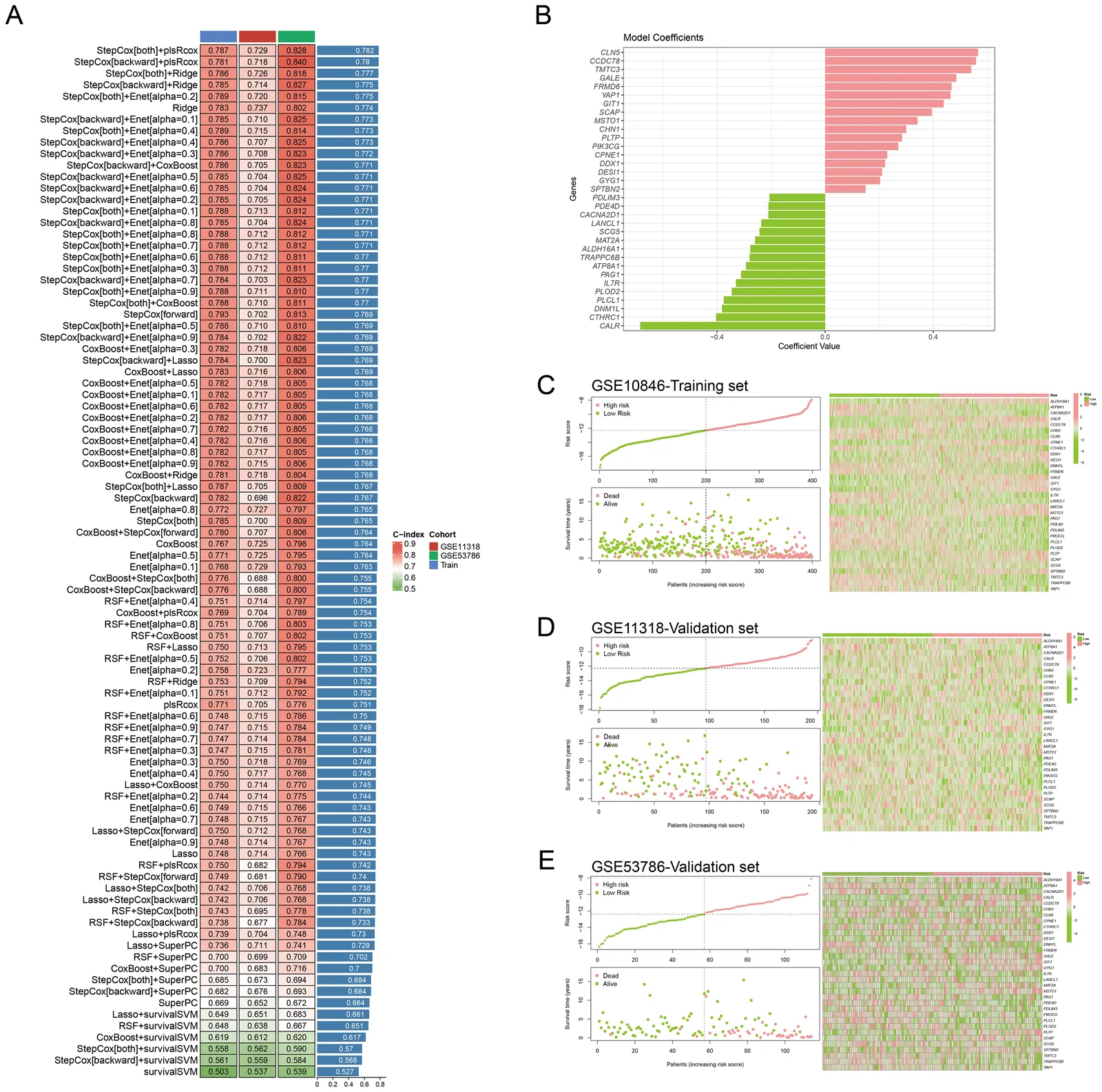
Construction of the PRS. **(A)** Comparison of 101 prognostic models; the model with the highest average C-index was selected as optimal. **(B)** Coefficients of the StepCox[both] + plsRcox model. **(C-E)** Risk plots for GSE10846, GSE11318, and GSE53786. Top left: risk score distribution. Bottom left: survival status distribution. Right: expression patterns of model genes.

### Validation of the prognostic performance of the PRS model

In the training cohort, overall survival analysis showed that patients in the low-risk group had significantly better survival than those in the high-risk group, with AUC values of 0.826, 0.845, and 0.848 for predicting 1-, 3-, and 5-year overall survival, respectively (**Figure 5A**). Similar findings were observed in the two validation cohorts, in which the low-risk group also showed better survival outcomes and the AUC values remained above 0.77, indicating consistent prognostic performance of the PRS across cohorts (**Figures 5B, C**).

**Figure 5:**
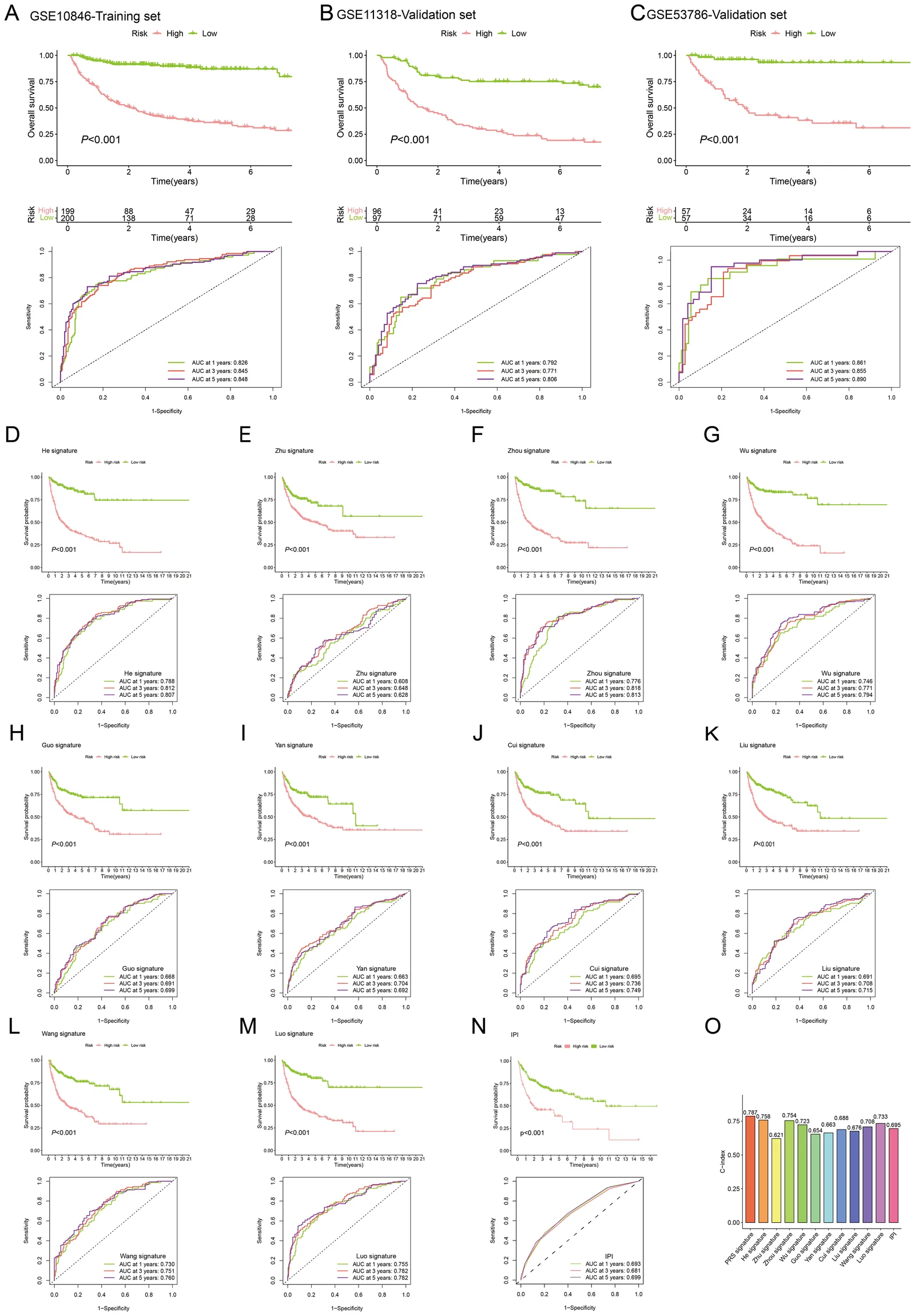
Evaluation of the prognostic performance of the PRS. **(A-C)** Kaplan-Meier survival curves and ROC curves for the training and validation cohorts (GSE10846, GSE11318, and GSE53786). **(D-N)** K-M survival curves and ROC curves of the 10 published DLBCL prognostic models and IPI. **(O)** Comparison of the C-index values between PRS, the 10 refitted published prognostic signatures, and the IPI.

To further evaluate its prognostic performance, the PRS was benchmarked against 10 previously published DLBCL prognostic gene signatures published between 2022 and 2025, as well as with the conventional IPI, under the same analytical framework in the GSE10846 cohort. Although these models also showed better outcomes in low-risk patients, their discriminatory performance was lower than that of the PRS, as reflected by lower AUC values (**Figures 5D–N**). Comparison of C-index values across all models further showed that the PRS achieved the highest C-index under this within-cohort benchmarking framework (**Figure 5O**).

### Construction of the nomogram

To improve the clinical applicability and individualized predictive value of PRS, we further integrated it with key clinical characteristics of DLBCL to construct a prognostic nomogram. Age, ECOG score, and stage differed significantly between the high- and low-risk groups (**Figure 6A**). The PRS was further confirmed as an independent prognostic factor for overall survival in both the training and the validation cohorts (**Figures 6B–D**). By combining the PRS with stage and ECOG score, the nomogram achieved a C-index of 0.805 (95% CI: 0.769-0.840; **Figure 6E**). Calibration curve, ROC, and C-index analyses demonstrated close agreement between the predicted and observed survival outcomes and indicated that the nomogram had better prognostic performance than the PRS alone or individual clinical characteristics (**Figures 6F–H**).

**Figure 6:**
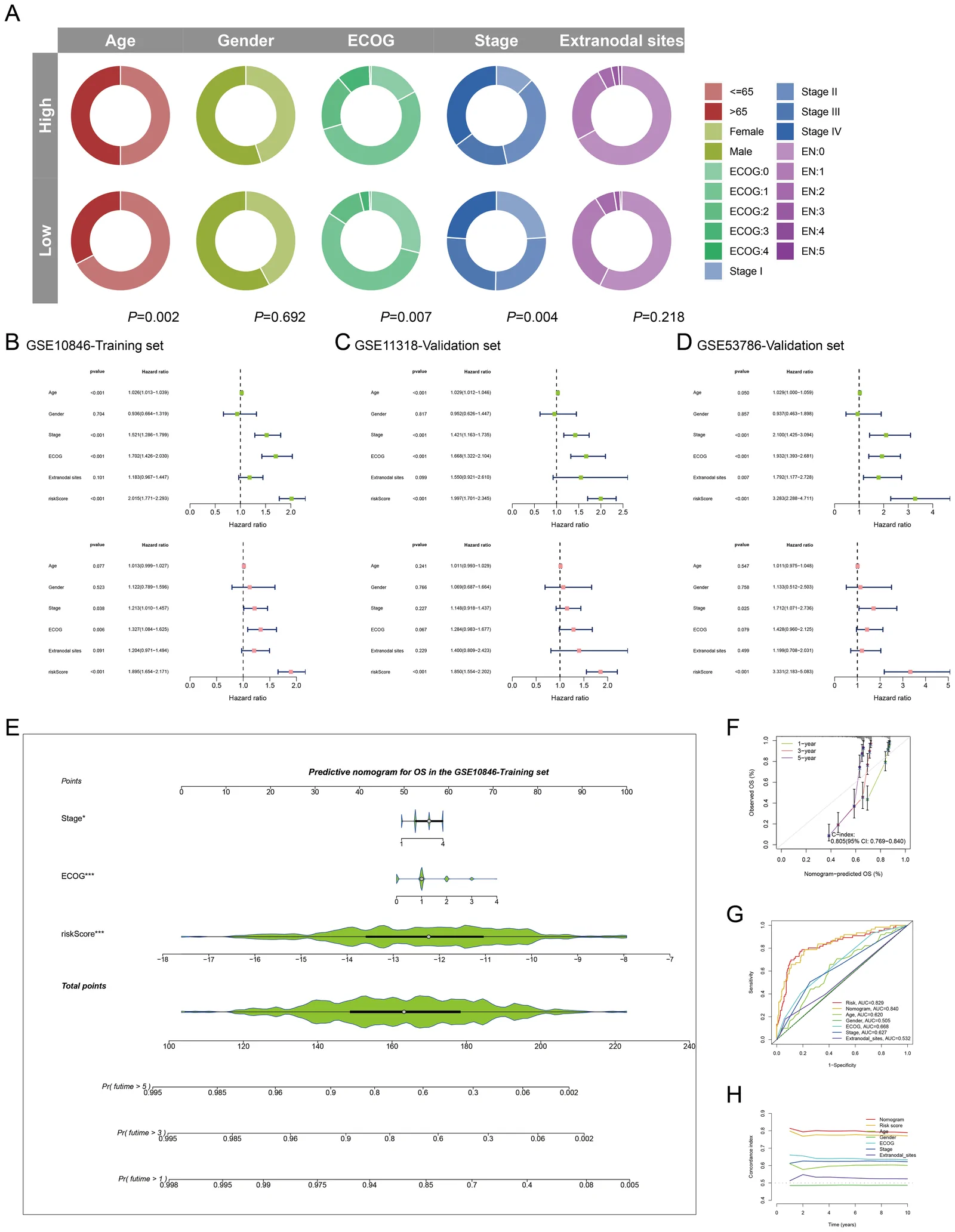
Construction and evaluation of the nomogram. **(A)** Circular plot of clinical characteristics in the high- and low-risk groups, showing differences in age, ECOG score, and stage. **(B-D)** Univariate and multivariate Cox regression analyses of clinical characteristics and PRS in the training and validation cohorts. **(E)** Nomogram integrating PRS and clinical characteristics. **(F)** Calibration curve. **(G, H)** ROC curve and time-dependent C-index curve of the nomogram, PRS, and clinical characteristics.

### Differential characteristics of the TME across risk groups

To characterize differences in the TIME across PRS risk groups, we compared TIME characteristics between the high- and low-risk groups. ESTIMATE analysis showed that low-risk patients had higher stromal scores and ESTIMATE scores, suggesting a higher proportion of non-tumor components and relatively lower tumor purity (**Figure 7A**). In addition, the 33 prognostic PDGEs comprising the PRS were significantly correlated with the infiltration levels of most immune cells, and the PRS itself was correlated with the abundance of eight immune cell types (**Figure 7B**). Specifically, naive B cell, M1 macrophages, M2 macrophages, regulatory T cells (Tregs), and NK cells were positively correlated with the PRS, whereas M0 macrophages, resting mast cells, and gamma delta T cells were negatively correlated with PRS (**Supplementary Figure S4**).

**Figure 7:**
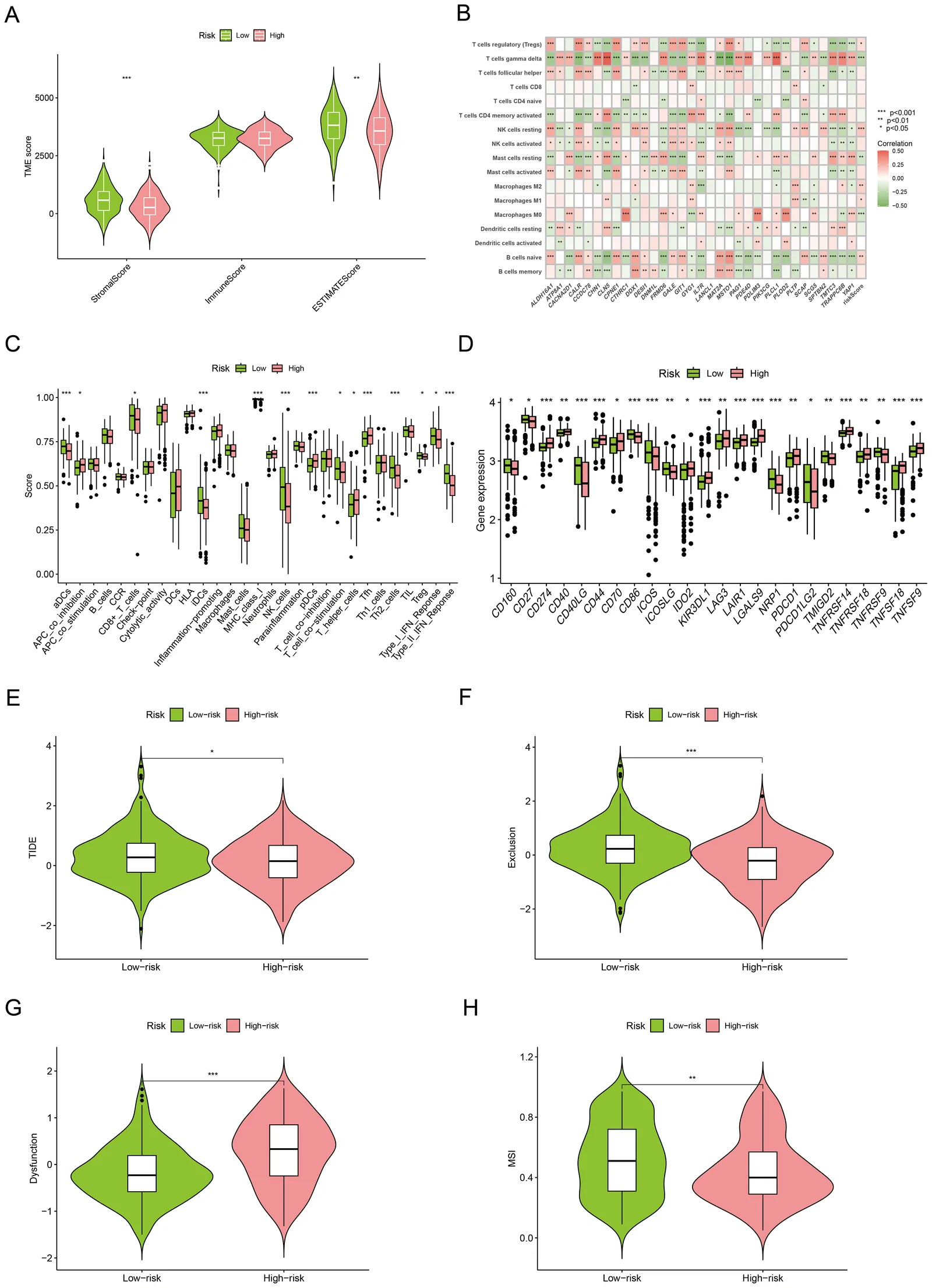
Immune microenvironment analysis associated with PRS. **(A)** Stromal score, immune score, and ESTIMATE score in the high- and low-PRS groups. **(B)** Heatmap of correlations between PRS, its 33 model genes, and immune cells. **(C)** Differences in 29 immune-related signatures between the high- and low-PRS groups. **(D)** Differences in the expression of immune checkpoint genes between the two risk groups. **(E-H)** Violin plots comparing TIDE, Exclusion, Dysfunction, and MSI scores between the two groups. (****P < 0.0001, ***P < 0.001, **P < 0.01; *P < 0.05).

Further analysis of the 29 immune-related signatures revealed significant differences in 14 signatures between the high- and low-risk groups (**Figure 7C**, *P* < 0.05), with 10 showing higher levels in the low-risk group. Among the 47 immune checkpoint genes evaluated, 24 were differentially expressed between the two groups, including 14 with higher expression in the high-risk group (**Figure 7D**). TIDE analysis further showed lower TIDE scores in the high-risk group, suggesting a lower predicted likelihood of tumor immune escape (**Figures 7E–H**). Together, these findings indicate distinct immune microenvironment characteristics between the two PRS risk groups, while the high-risk group may have a greater predicted potential to benefit from immune checkpoint inhibitor (ICI) therapy; however, this possibility requires further validation in ICI-treated DLBCL cohorts.

### Analysis of predicted drug sensitivity

To explore PRS-associated differences in predicted drug-response profiles, IC50 values for 198 compounds were estimated for each patient using the GDSC database, and differences in predicted IC50 values between the high- and low-risk groups, together with their correlations with PRS scores, were analyzed. Ten compounds, including 5-FU, cisplatin, dabrafenib, dactinomycin, epirubicin, I_BET_762, irinotecan, leflunomide, selumetinib, and temozolomide, showed lower predicted IC50 values in high-risk patients, suggesting greater predicted sensitivity to these agents in the high-risk group (**Figures 8A–J**). Notably, the predicted IC50 values of all 10 compounds were significantly negatively correlated with PRS scores (**Supplementary Figures S5A–J**).

**Figure 8:**
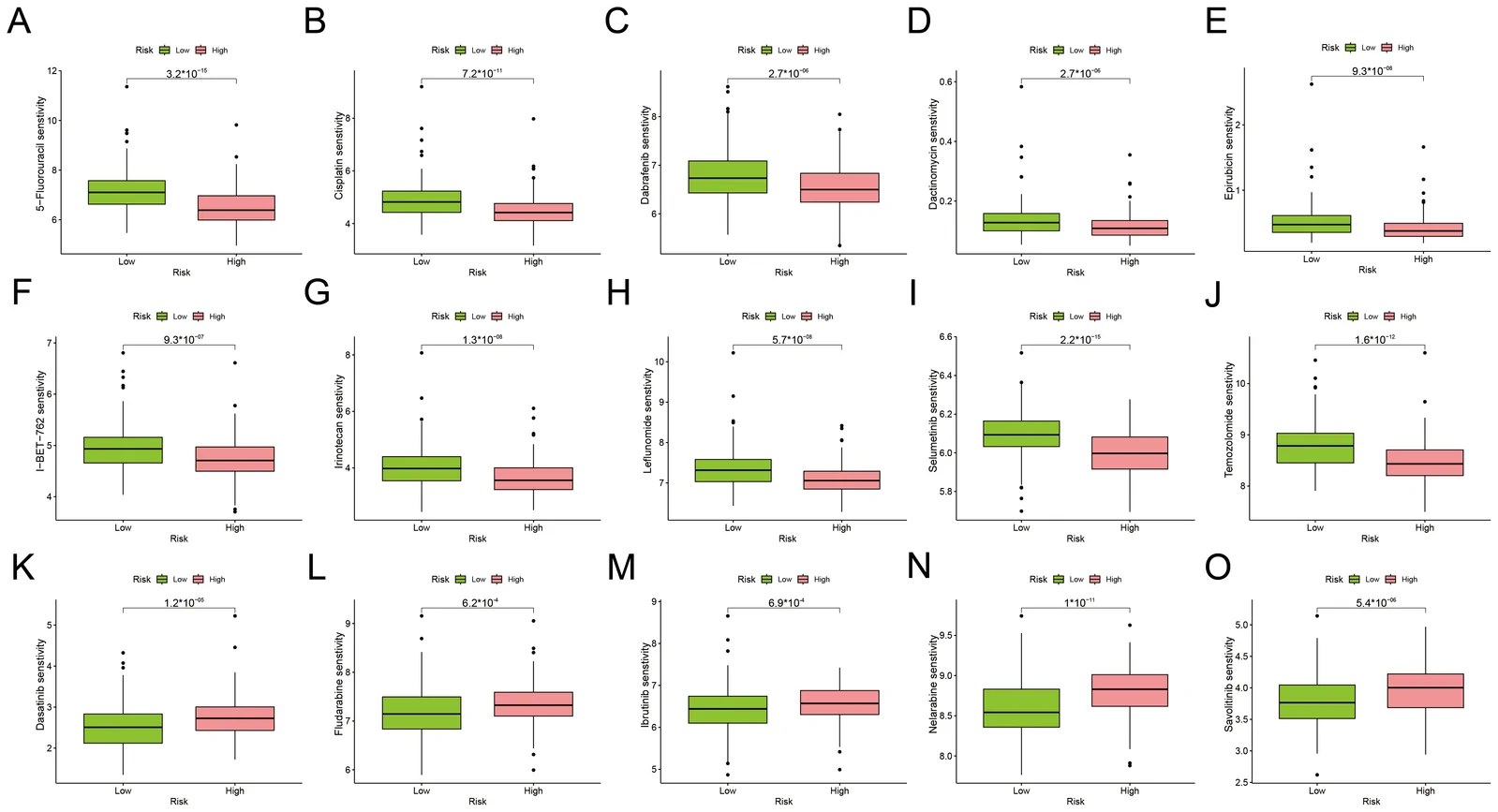
Analysis of predicted drug sensitivity according to PRS. **(A-J)** Compounds applicable showing lower predicted IC50 values in the high-PRS group: 5-FU, cisplatin, dabrafenib, dactinomycin, epirubicin, I_BET_762, irinotecan, leflunomide, selumetinib, temozolomide. **(K-O)** Compounds showing lower predicted IC50 values in the low-PRS group: dasatinib, fludarabine, ibrutinib, nelarabine, and savolitinib.

In contrast, five compounds, including dasatinib, fludarabine, ibrutinib, nelarabine, and savolitinib, showed higher predicted IC50 values in high-risk patients, suggesting relatively greater predicted sensitivity to these agents in the low-risk group. Their predicted IC50 values were also significantly positively correlated with PRS scores (**Figures 8K–O**, **Supplementary Figures S5K–O**). These findings indicate distinct predicted drug-response patterns between the two PRS risk groups.

### WGCNA and MR Analysis

To investigate differences in gene co-expression patterns between the high- and low-risk groups, WGCNA was performed on DEGs. A weighted co-expression network was constructed to identify gene modules highly associated with the risk group. WGCNA identified 10 gene modules with distinct expression patterns (**Figure 9A**, **Supplementary Figures S6A, B**). Among them, the blue module showed the strongest association with risk grouping and was identified as the key module most closely associated with the PRS risk group (**Figures 9B, C**). Key genes in the blue module are listed in **Supplementary Table 8**.

**Figure 9:**
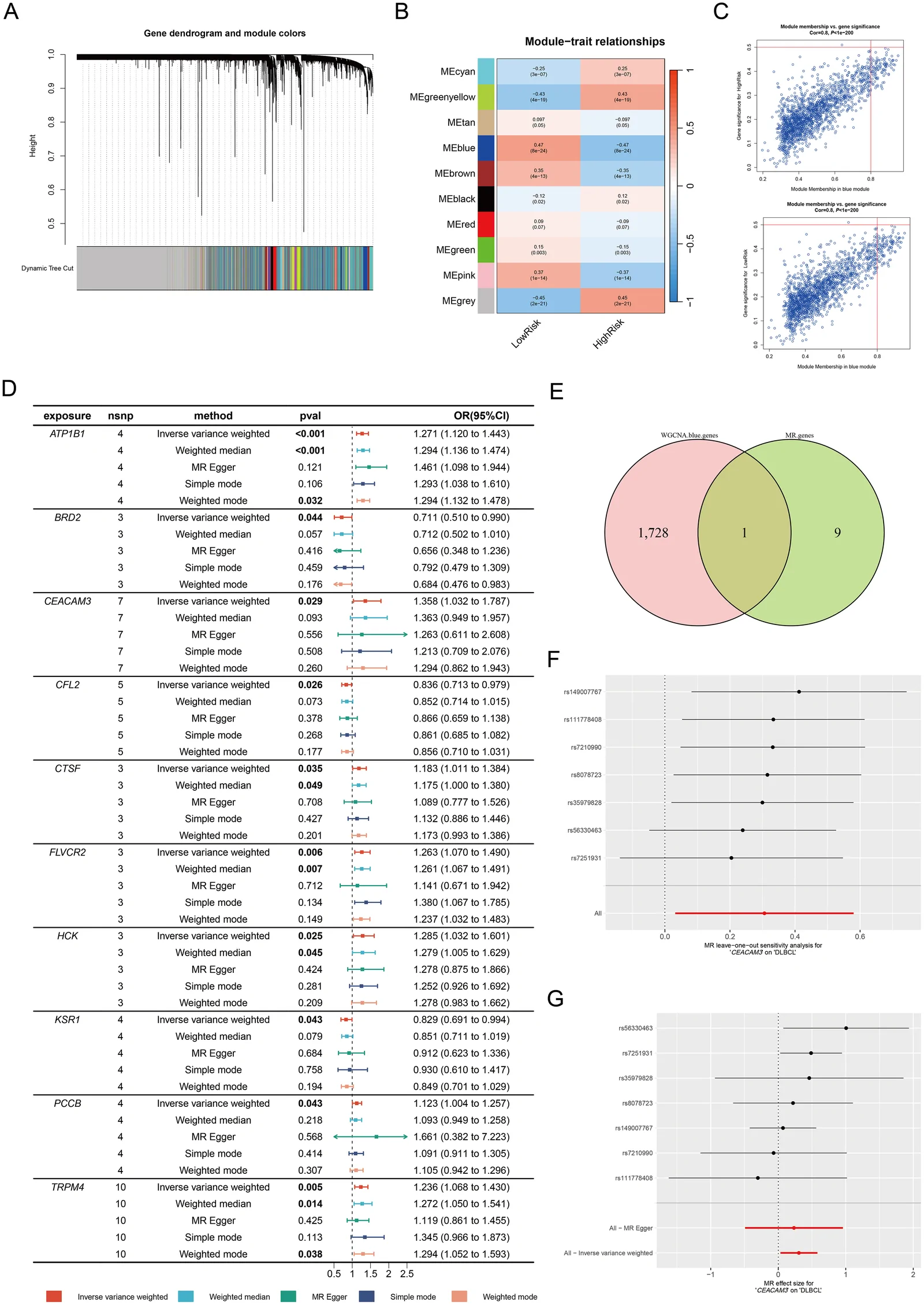
WGCNA and MR Analyses. **(A)** Gene clustering dendrogram showing modules identified by WGCNA, with different colors representing different modules **(B)** Heatmap of module-trait relationships showing the associations between co-expression modules and PRS risk groups **(C)** Scatter plot showing the association between module membership and gene significance in the key blue module. **(D)** Forest plot of MR analyses showing 10 genes with nominal evidence of potential associations with DLBCL (*P* < 0.05) **(E)** Venn plot showing the intersection between genes in the WGCNA blue module and the 10 genes showing nominal MR associations with DLBCL. **(F)** Leave-one-out sensitivity analysis for the MR assessment of *CEACAM3*. **(G)** Forest plot showing the causal effect estimates and 95% confidence intervals for individual IVs used in the MR analysis of *CEACAM3* expression on DLBCL risk.

MR analysis identified 10 genes, including *ATP1B1*, *BRD2*, *CEACAM3*, *CFL2*, *CTSF*, *FLVCR2*, *HCK*, *KSR1*, *PCCB*, and *TRPM4*, showing nominal evidence of potential causal associations with DLBCL (**Figure 9D**, **Supplementary Tables 9**, **10**, *P* < 0.05). However, none of these associations remained significant after FDR correction. By integrating the WGCNA blue module with the nominal MR findings, *CEACAM3* was identified as a key candidate gene (**Figures 9E–G**, **Supplementary Figures S6C, D**) associated with the PRS risk phenotype for further functional investigation.

### Functional characterization of *CEACAM3* in DLBCL

*CEACAM3* expression was higher in DLBCL tissues than in normal tissues and was also elevated in the C1 subgroup (**Supplementary Figures S7A, B**). Survival analysis further showed that patients with low *CEACAM3* expression had better prognosis than those with high *CEACAM3* expression (**Supplementary Figure S7C**). To select appropriate DLBCL cell lines for functional validation, we first examined the basal protein expression of CEACAM3 in five DLBCL cell lines, including SU-DHL-4, TOLEDO, TMD8, OCI-LY3, and OCI-LY10, by western blotting. Among the five cell lines examined, SU-DHL-4 cells exhibited the highest *CEACAM3* protein expression, whereas TMD8 cells showed relatively low expression (**Figure 10A**). Therefore, SU-DHL-4 cells were selected for *CEACAM3* knockdown experiments, and TMD8 cells were selected for *CEACAM3* overexpression experiments. We first established stable pooled SU-DHL-4 cell populations with *CEACAM3*-targeting sgRNA, which showed reduced *CEACAM3* expression compared with the sgRNA control group (**Figure 10B**). Functionally, *CEACAM3* knockdown significantly inhibited cell growth, as shown by reduced proliferative activity in the CCK-8 assay (**Figure 10C**). Representative images showed that *CEACAM3* knockdown markedly reduced the number of invading SU-DHL-4 cells compared with the control group (**Figure 10D**), which was further confirmed by quantitative analysis (**Figure 10E**). Consistently, TUNEL staining showed a pronounced increase in apoptosis after *CEACAM3* knockdown, reflected by a higher proportion of TUNEL-positive cells (**Figures 10F, G**). Together, these results indicate that stable pooled *CEACAM3* knockdown promotes apoptosis and suppresses proliferation and invasion in SU-DHL-4 cells.

**Figure 10:**
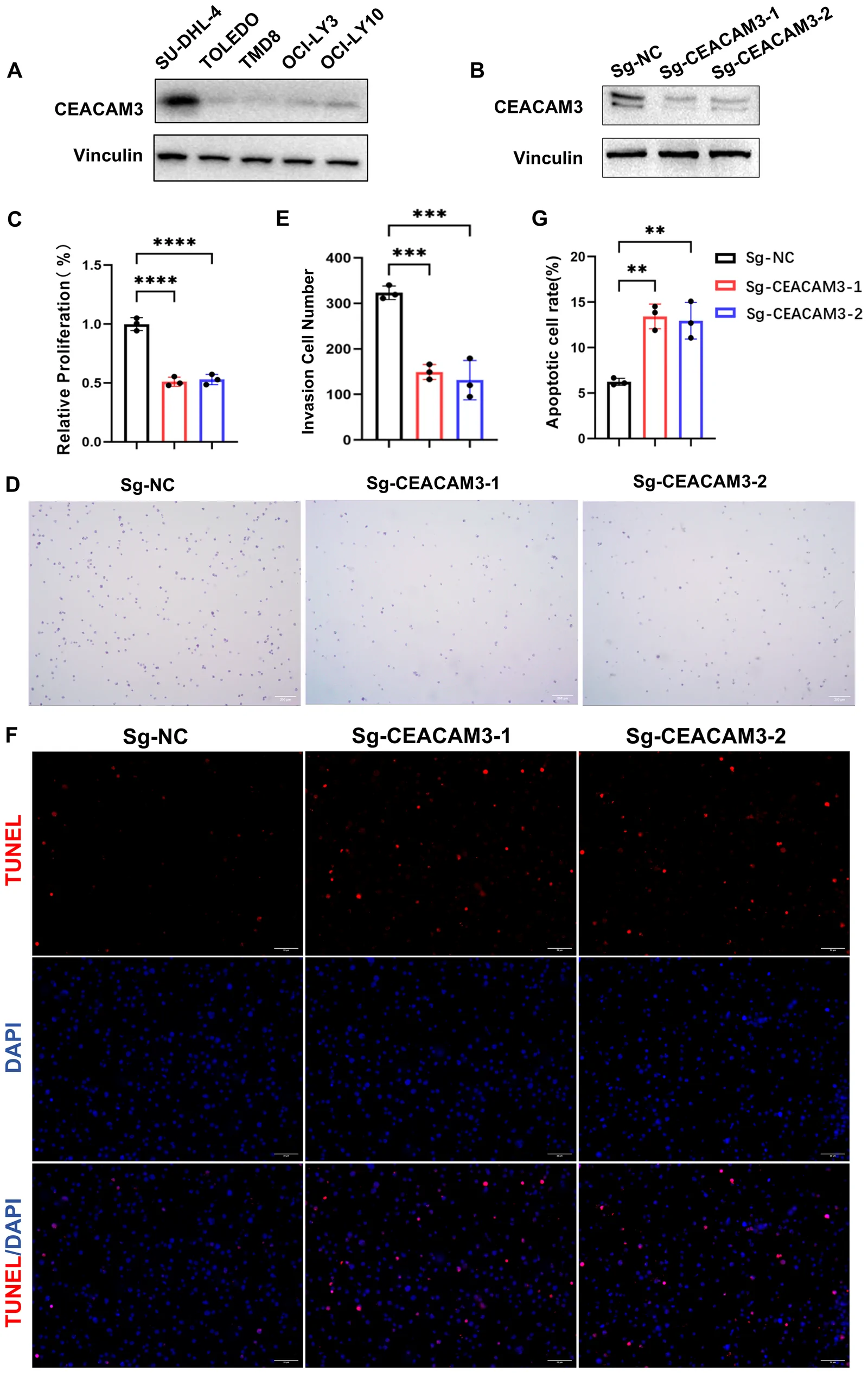
Stable pooled *CEACAM3* knockdown suppresses proliferation and invasion and promotes apoptosis in SU-DHL-4 cells. **(A)** Detection of CEACAM3 protein expression in different DLBCL cells. **(B)** Western blot analysis showing reduced CEACAM3 protein expression in *CEACAM3*-knockdown SU-DHL-4 cells compared with negative control cells. Vinculin was used as the loading control. **(C)** CCK-8 assay showing that *CEACAM3* knockdown significantly inhibited the proliferative capacity of SU-DHL-4 cells. (n=3). **(D)** Representative images of crystal violet-stained invaded cells showing reduced invasion after *CEACAM3* knockdown. Scale bar=200 μm. **(E)** Quantification of invaded cells showing that *CEACAM3* knockdown significantly decreased the number of invading SU-DHL-4 cells. (n=3). **(F)** Representative TUNEL staining images showing increased apoptosis in *CEACAM3*-knockdown SU-DHL-4 cells. Scale bar=20 μm. **(G)** Quantification of apoptotic cells showing that *CEACAM3* knockdown significantly increased apoptosis. (n=3). **P < 0.01, ***P < 0.001, ****P < 0.0001.

To further confirm the biological role of *CEACAM3*, a complementary gain-of-function experiment was performed in TMD8 cells. Western blotting confirmed the successful expression of FLAG-tagged *CEACAM3* in the stable *CEACAM3* overexpressing TMD8 cells (**Figure 11A**). Representative images from the Matrigel-coated Transwell assay and the corresponding quantitative analysis demonstrated that *CEACAM3* overexpression significantly increased the invasive capacity of TMD8 cells (**Figures 11B, C**). The CCK-8 assay showed that *CEACAM3* overexpression significantly enhanced TMD8 cell proliferation compared with the control group (**Figure 11F**). In contrast, TUNEL staining revealed a significant reduction in the proportion of apoptotic cells following *CEACAM3* overexpression (**Figures 11D, E**). To further evaluate the effect of *CEACAM3 in vivo*, TMD8 cells were used to establish subcutaneous xenograft models in NPG mice. At 15 days after inoculation, the excised xenograft tumors derived from *CEACAM3* overexpressing TMD8 cells were visibly larger than those derived from the control cells (**Figure 11G**). Consistently, the final tumor weight was significantly higher in the *CEACAM3* overexpression group than in the control group (**Figure 11H**). Dynamic monitoring of tumor growth further showed that *CEACAM3* overexpression accelerated xenograft growth and resulted in significantly greater tumor volumes at the indicated time points (**Figure 11I**). Furthermore, immunohistochemical staining showed that xenograft tumors in the *CEACAM3* overexpression group had a significantly higher percentage of Ki-67 positive cells than those in the control group (**Figures 11J, K**). Collectively, these findings demonstrate that *CEACAM3* promotes DLBCL cell proliferation and invasion, suppresses apoptosis, and enhances tumor growth *in vivo*, further supporting the tumor-promoting role of *CEACAM3* in DLBCL.

**Figure 11:**
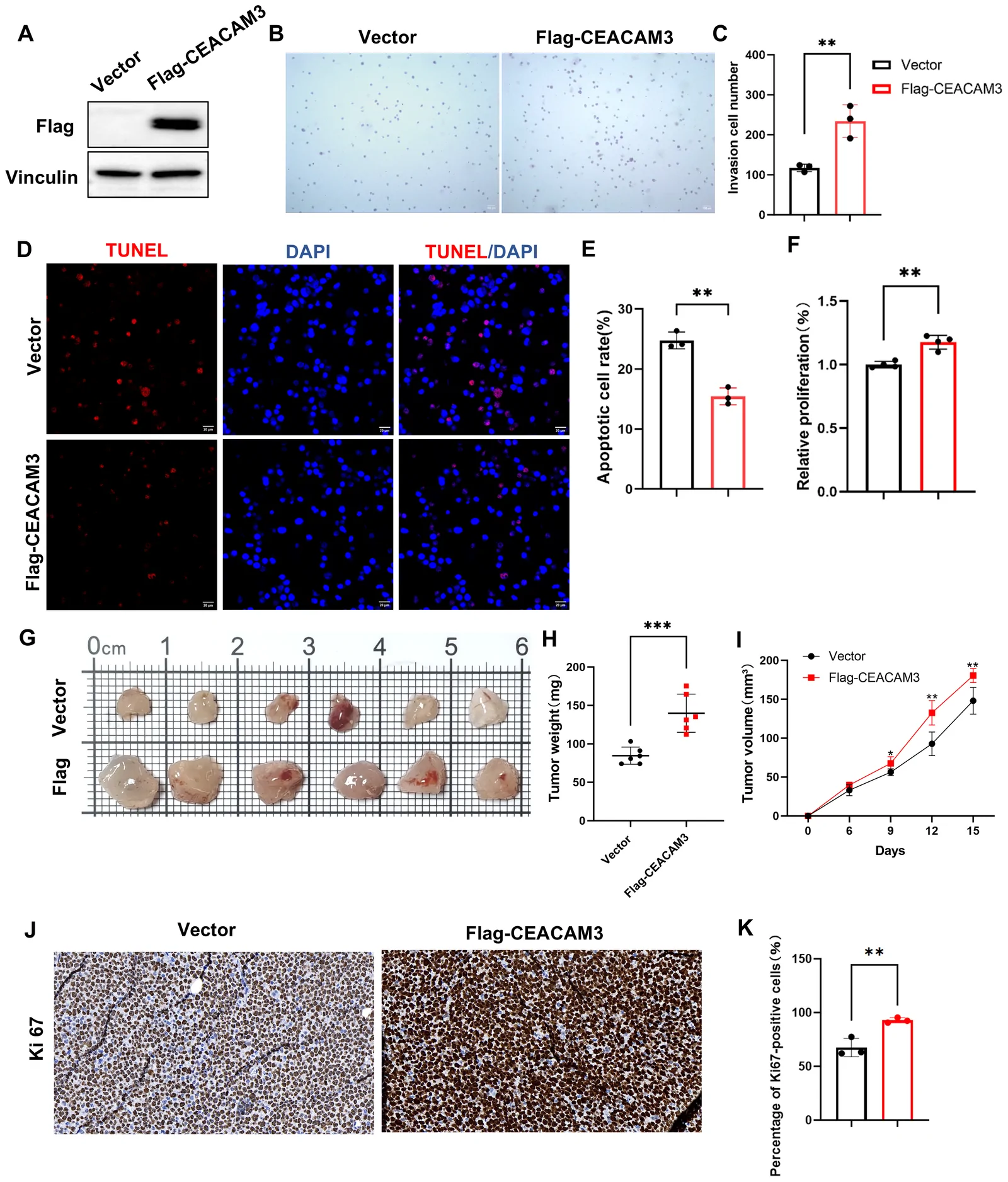
Stable *CEACAM3* overexpression promotes proliferation and invasion, suppresses apoptosis, and enhances xenograft tumor growth in TMD8 cells. **(A)** Western blot analysis confirming FLAG-tagged CEACAM3 expression in stable *CEACAM3* overexpressing TMD8 cells compared with the control cells. Vinculin was used as the loading control. **(B)** Representative images of crystal violet-stained invaded cells showing increased invasion following *CEACAM3* overexpression. Scale bar=100 μm. **(C)** Quantification of invaded cells showing that *CEACAM3* overexpression significantly increased the number of invading TMD8 cells (n=3). **(D)** Representative TUNEL staining images showing decreased apoptosis in *CEACAM3* overexpressing TMD8 cells. Scale bar=20 μm. **(E)** Quantification of TUNEL-positive cells showing that *CEACAM3* overexpression significantly suppressed TMD8 cell apoptosis (n=3). **(F)** CCK-8 assay showing that *CEACAM3* overexpression significantly promoted TMD8 cell proliferation (n=3). **(G)** Representative images of xenograft tumors excised from mice in the control and *CEACAM3* overexpression groups on day 15 after tumor-cell inoculation (n=6). **(H)** Final xenograft tumor weights showing a significant increase in the *CEACAM3* overexpression group (n=6). **(I)** Xenograft tumor growth curves showing that *CEACAM3* overexpression accelerated tumor growth *in vivo* (n=6). **(J)** Representative images of Ki-67 immunohistochemical staining in xenograft tumors. Scale bar=20 μm. **(K)** Quantification of Ki-67-positive cells showing a significantly higher Ki-67 positive fraction in xenograft tumors derived from *CEACAM3* overexpressing TMD8 cells (n=6). **P < 0.01 and ***P < 0.001.

## Discussion

Palmitoylation is a crucial and dynamically reversible post-translational protein modification that regulates protein localization and function within cells (38). However, its molecular role and clinical relevance in DLBCL remain unclear. In this study, we systematically investigated PRGs in DLBCL and developed a PRG-based prognostic model to identify new molecular subtypes, characterize immune microenvironmental heterogeneity, and explore potential therapeutic targets.

Using 99 prognostic PDGEs, NMF clustering identified two molecular subtypes of DLBCL, C1 and C2. These subtypes differed in prognosis, immune microenvironment characteristics, and core biological functions. Patients in the C2 subtype had better survival outcomes, accompanied by higher immune cell infiltration and greater activity of immune-related pathways. These differences in the immune microenvironment may partly contribute to the more favorable clinical outcome observed in C2.

We further assessed the prognostic value of PRGs by integrating 10 machine learning algorithms and constructing a PRS model composed of 33 PRGs. Among the 101 modeling approaches evaluated, StepCox[both] + plsRcox achieved the highest C-index across the training and validation cohorts and was selected as the final model. The PRS showed stable prognostic performance across cohorts and demonstrated favorable performance in the within-cohort benchmarking analysis, with higher C-index and AUC values than the 10 refitted published DLBCL prognostic signatures and the established IPI (2, 39–47). These findings suggest that palmitoylation-related transcriptional patterns may provide prognostic information beyond that captured by existing models. Because the clinical value of a prognostic model also depends on its usefulness for individualized risk assessment (48), we further integrated the PRS with key clinical variables to construct a prognostic nomogram. The nomogram showed better prognostic performance than the PRS alone and provided a more comprehensive approach to individualized risk prediction.

The TME plays an important role in tumor progression, immune escape, and response to therapy (49, 50). We therefore compared TME characteristics between the two risk groups. The low-risk group showed higher stromal and ESTIMATE scores, lower tumor purity, and greater activity of immune-related pathways, indicating marked differences in the immune contexture between the two groups. This may help explain the survival difference between the two groups. The PRS was also significantly correlated with the infiltration abundance of eight immune cell types. In particular, M2 macrophages and Tregs were positively correlated with the PRS. Both cell types have well-established immunosuppressive functions and may contribute to an immune-evasive microenvironment in patients with higher PRS (51, 52). At the same time, naive B cell and M1 macrophages, which may exert antitumor effects in certain contexts, were also positively correlated with the PRS (53, 54). These seemingly divergent associations highlight the complexity of the DLBCL immune microenvironment and suggest that a high PRS may reflect an altered immune state rather than a simple reduction in immune infiltration. By contrast, macrophages M0, resting mast cells, and γδ T cells were negatively correlated with the PRS, suggesting that the low-PRS group may retain stronger innate immune surveillance and earlier anti-tumor responses (55–57). TIDE analysis further suggested that patients in the high-PRS group may have a greater likelihood of responding to ICI therapy. Taken together, these findings support a close association between palmitoylation-related transcriptional patterns and the immune contexture of DLBCL. Drug sensitivity analysis further revealed distinct predicted response profiles between the two PRS risk groups, suggesting potential differences in therapeutic sensitivity. However, these findings were based on computational predictions and require further validation in experimental models and clinical cohorts before PRS can be used to guide treatment stratification.

To further explore the potential relationship between PDGEs and DLBCL risk, we performed MR analysis and identified 10 PDGEs showing nominal evidence of potential causal associations with DLBCL. *ATP1B1* has been reported to promote the proliferation and invasion of DLBCL cells (58). *BRD2*, a member of the BET family, participates in cell-cycle regulation, chromatin remodeling, and transcriptional control and regulates multiple oncogenes, making it a potential therapeutic target in cancer (59–61). *CFL2* regulates cytoskeletal dynamics and has been implicated in tumor cell migration and invasion, although its role in DLBCL remains unclear (62–65). *CTSF* is a lysosomal cysteine protease involved in intracellular protein degradation and autophagy and has also been associated with tumor progression and immune regulation through processes including antigen presentation and apoptosis (66, 67). *FLVCR2*, a member of the major facilitator superfamily, has been implicated in calcium transport and heme uptake. It has been reported as a progression-related biomarker in prostate cancer and acute myeloid leukemia and was identified in our analysis as a potential risk-related gene in DLBCL, although its specific role remains unclear (68–71). *HCK* is a Src family non-receptor tyrosine kinase that promotes malignant B-cell proliferation and survival through signaling pathways involving Bruton’s tyrosine kinase (BTK) and has been proposed as a potential therapeutic target in DLBCL (72–74). *KSR1* is a scaffold protein in the RAS/MAPK pathway with context-dependent functions and may exert either tumor-promoting or tumor-suppressive effects (75). *PCCB* mutations are known to cause propionic acidemia, while emerging evidence suggests that *PCCB* may also participate in TME remodeling through the regulation of cellular and immune metabolism (76, 77). *TRPM4* is a calcium-sensitive ion channel that is highly expressed in DLBCL and has been associated with poor prognosis (78).

In our study, WGCNA linked *CEACAM3* to the PRS-defined risk phenotype, while MR analysis provided nominal genetic evidence for its potential association with DLBCL. Integrating these findings identified *CEACAM3* as a key candidate for further investigation. It promotes neutrophil phagocytosis and induces a strong inflammatory response (79). Previous studies have also shown that *CEACAM3* is mainly enriched in granulocytes in the TME, particularly neutrophils (80), where it enhances phagocytosis and triggers inflammatory responses after pathogen invasion (81). *CEACAM3* can also be phosphorylated by Src family protein tyrosine kinases during phagocytic signaling, a family that includes HCK, another PRG identified in our analysis (82). To examine its direct effect on tumor cells, we performed both loss-of-function and gain-of-function experiments in DLBCL models. *CEACAM3* knockdown in SU-DHL-4 cells suppressed proliferation and invasion and promoted apoptosis, whereas *CEACAM3* overexpression in TMD8 cells enhanced proliferation and invasion, reduced apoptosis, and accelerated xenograft growth *in vivo*. These complementary findings provide stronger support for the tumor-promoting role of *CEACAM3* in DLBCL and further support its potential value as a therapeutic target. However, the downstream molecular pathways through which *CEACAM3* drives these malignant phenotypes remain to be clarified.

This study has several limitations. First, the PRS was developed using retrospective data and still requires prospective validation. Second, the immunotherapy- and drug sensitivity-related findings were derived from computational analyses and should be further validated in clinically annotated treatment cohorts and experimental models, particularly because some of the identified compounds are not routinely used for DLBCL. Third, the MR analysis was based on whole-blood eQTL data, which may not fully capture B-cell-specific regulatory effects, and future replication using lymphoid- or B-cell-specific eQTL resources is warranted. Finally, the PRG set used in this study was derived from GeneCards and represents a broad palmitoylation-related gene set rather than exclusively experimentally validated S-palmitoylated proteins. Further investigation of the biological mechanisms underlying the PRS, as well as the downstream signaling pathways of *CEACAM3*, may provide additional insights into their roles in DLBCL progression and strengthen the biological basis of the model.

## Conclusion

In this study, we developed a PRG-based prognostic model for DLBCL and evaluated its prognostic performance across multiple cohorts. The model effectively stratified patients according to prognosis and showed potential value in characterizing predicted drug sensitivity and immunotherapy-related features. In addition, functional experiments indicated that *CEACAM3* promotes tumor cell proliferation and invasion, suppresses apoptosis, and enhances tumor growth, supporting its potential as a therapeutic target in DLBCL.

## Data Availability

The original contributions presented in the study are included in the article/**Supplementary Material**. Further inquiries can be directed to the corresponding authors.
